# Hospitalization for ischemic stroke was affected more in independent cases than in dependent cases during the COVID-19 pandemic: An interrupted time series analysis

**DOI:** 10.1371/journal.pone.0261587

**Published:** 2021-12-17

**Authors:** Hiroyuki Nagano, Jung-ho Shin, Tetsuji Morishita, Daisuke Takada, Susumu Kunisawa, Kiyohide Fushimi, Yuichi Imanaka

**Affiliations:** 1 Department of Healthcare Economics and Quality Management, Graduate School of Medicine, Kyoto University, Kyoto City, Japan; 2 Department of Health Policy and Informatics, Graduate School of Medicine, Tokyo Medical and Dental University, Yushima, Bunkyo-ku, Tokyo, Japan; Hospital Dr. Rafael A. Calderón Guardia, CCSS, COSTA RICA

## Abstract

**Background:**

The pandemic of the coronavirus disease 2019 (COVID-19) has affected health care systems globally. The aim of our study is to assess the impact of the COVID-19 pandemic on the number of hospital admissions for ischemic stroke by severity in Japan.

**Methods:**

We analysed administrative (Diagnosis Procedure Combination—DPC) data for cases of inpatients aged 18 years and older who were diagnosed with ischemic stroke and admitted during the period April 1 2018 to June 27 2020. Levels of change of the weekly number of inpatient cases with ischemic stroke diagnosis after the declaration of state of emergency were assessed using interrupted time-series (ITS) analysis. The numbers of patients with various characteristics and treatment approaches were compared. We also performed an ITS analysis for each group (“independent” or “dependent”) divided based on components of activities of daily living (ADL) and level of consciousness at hospital admission.

**Results:**

A total of 170,294 cases in 567 hospitals were included. The ITS analysis showed a significant decrease in the weekly number of ischemic stroke cases hospitalized (estimated decrease: −156 cases; 95% confidence interval (CI): −209 to −104), which corresponds to −10.4% (95% CI: −13.6 to −7.1). The proportion of decline in the independent group (−21.3%; 95% CI: −26.0 to −16.2) was larger than that in the dependent group (−8.6%; 95% CI: −11.7 to −5.4).

**Conclusions:**

Our results show a marked reduction in hospital admissions due to ischemic stroke after the declaration of the state of emergency for the COVID-19 pandemic. The independent cases were affected more in proportion than dependent cases.

## Introduction

The coronavirus disease 2019 (COVID-19), first recognized in Wuhan, China, in early December 2019 [1], spread globally in 2020. In Japan, the daily number of new COVID-19 infections increased dramatically from late March and April 2020 [2]. In response, the Japanese government declared a state of emergency on April 7 2020 for seven prefectures and broadened the declaration to all 47 prefectures on April 16 2020 [3].

In the world, stroke is the second leading cause of death and a major cause of disability [4]. Ischemic stroke represents about 70% of all strokes in Japan and globally [5, 6]. Treatments for ischemic stroke, especially intravenous thrombolysis and endovascular intervention, are time-sensitive and should be initiated as quickly as possible [7]. Therefore, patients with symptoms suggesting ischemic stroke should be evaluated by medical staff immediately. However, movement restrictions and the fear of COVID-19 transmission in hospitals during the COVID-19 pandemic may have led to hesitation by individuals to seek medical services, resulting in delayed access and a higher risk of severe illness [8, 9]. Although previous studies have shown a decline in hospital admissions for stroke during the COVID-19 pandemic [10, 11], not many studies have focused on finding the characteristics of stroke patients who were impacted by the COVID-19 pandemic [12]. Therefore, we performed a retrospective cohort study using a large-scale Japanese database. Our study sought to provide an evaluation of the impact of the COVID-19 pandemic on the number of hospital admissions for ischemic stroke by severity in Japan. We also investigated the change of proportion of cases receiving intravenous thrombolysis or endovascular intervention for ischemic stroke during hospitalization.

## Methods

### Data source

We used Diagnosis Procedure Combination (DPC) data from the database of the DPC research group, which is funded by the Ministry of Health, Labour and Welfare, Japan. The DPC/per-diem payment system (PDPS) is a Japanese prospective payment system applied to acute care hospitals. In 2018, a total of 1,730 hospitals used the DPC/PDPS, accounting for 54% (482,618 out of 891,872) of all the general hospital beds in Japanese hospitals [13, 14]. The DPC data consists of discharge summaries, which include the International Classification of Diseases 10^th^ Revision (ICD-10) codes classifying main diagnosis, the trigger diagnosis, the most and second-most medical-resource-intensive diagnoses, up to 10 comorbidities, and up to 10 complications during hospitalization [15]. The database also includes the following patient details: age, sex, body mass index (BMI), medical procedures, daily records of drug administration, activities of daily living (ADL) according to the components of the Barthel index at hospital admission and at discharge, discharge destination and level of consciousness based on the Japan Coma Scale (JCS) at hospital admission and at discharge. In the JCS, code 0 refers to a patient who is alert, while codes 1 to 3 refer to a patient who awake without any stimulation, codes 10 to 30 refer to a patient who can be aroused by some stimulation, and codes 100 to 300 refer a patient who cannot be aroused by any stimulation.

### Study population and study subjects

We selected cases in which patients aged 18 years and older were hospitalized due to ischemic stroke between April 1 2018, and June 27 2020. For evaluation, we included all cases including hospital inpatients that provided DPC data continuously during the period of the study. Ischemic stroke was identified by ICD-10 code I63.x as the diagnosis that caused the hospital admission. Inpatient cases for which components of the Barthel index at hospital admission were not recorded were excluded. We defined the severity of ischemic stroke according to ADL and level of consciousness at hospital admission and divided the study population into two groups, “independent” or “dependent”. We defined “independent” as inpatient cases for which all components of the Barthel index at hospital admission were independent and JCS at hospital admission was “0”. We defined the other cases as “dependent”. The primary outcome was a change in the level of the weekly number of ischemic stroke hospitalizations before and after the declaration of first state of emergency in Japan. We hypothesized that the COVID-19 pandemic would affect the weekly number of cases after the 106^th^ week of the study period (between April 5 and April 11 2021), since the Japanese government announced the state of emergency on this week (April 7 2020) [3]. The secondary outcome was a change in the proportion of cases receiving intravenous thrombolysis or endovascular intervention for ischemic stroke hospitalizations.

### Statistical analysis

Continuous variables (such as age) are expressed as median values with interquartile range (IQR). Comparisons of the continuous variables for the two groups were performed using the Mann-Whitney U test. Categorical data (such as sex) were compared using a chi-square test with Yate’s correction. All statistical analyses were performed with R version 3.6.0 (R Foundation for Statistical Computing, Vienna, Austria). A two-sided p-value < 0.05 was considered statistically significant, and 95% confidence intervals (CI) were used for the outcomes.

### Interrupted time series analysis for change in case numbers

An interrupted time series (ITS) analysis was performed to evaluate the impact of the COVID-19 pandemic on the weekly number of inpatient cases [16]. The intervention time point was the 106^th^ week, when the Japanese government announced the state of emergency. The dependent variables were the weekly numbers of inpatient cases for ischemic stroke. The independent variables were weekly numbers and dichotomous dummy variables indicating whether the week was in the pre-intervention or the post-intervention period. Seasonality was considered by including harmonic terms (sines and cosines) [16]. We also performed an ITS analysis for each group (“independent” or “dependent”).

### Interrupted time series analysis for changes in weekly proportions of cases receiving intravenous thrombolysis or endovascular intervention

The changes in weekly proportions of cases receiving intravenous thrombolysis or endovascular intervention were also assessed by the ITS analysis. The dependent variables were the weekly numbers of inpatient cases for ischemic stroke receiving intravenous thrombolysis or endovascular intervention. The independent variables were weekly numbers and dichotomous dummy variable indicating whether the week was in the pre-intervention or the post-intervention period. “Ischemic stroke cases in the corresponding week” was handled as an offset term. Seasonality was considered by including harmonic terms (sines and cosines). The validity of the model was assessed by the using the correlograms (autocorrelation and partial autocorrelation functions) and residual plots.

### Ethical consideration

This study was approved by the Ethics Committee, Graduate School of Medicine, Kyoto University (approval number: R0135), and conducted in accordance with the Ethical Guidelines for Medical and Health Research Involving Human Subjects of the Ministry of Health, Labour and Welfare, Japan. According to these guidelines, written informed consent was waived for this research as it did not utilize human biological specimens and any information utilized in the research has been anonymized.

## Results

A total of 170,294 cases in 567 hospitals were included (S1 Fig). The total numbers of inpatient cases, characteristics, and time-sensitive treatment approaches before and after the declaration of state of emergency are presented in Table 1. The median of length of hospital stay decreased after the declaration of state of emergency, especially among the inpatient cases who transferred to other facilities (S1 and S2 Tables).

**Table 1:** The total number, characteristics, and time-sensitive treatment approaches of inpatient cases with ischemic stroke before and after the declaration of state of emergency.

	All hospitalized cases	Before the declaration of state of emergency	After the declaration of state of emergency	p
Number of cases	170,294	154,039	16,255	
Age, y, median [IQR]	78 [69–85]	78 [69–85]	78 [70–85]	0.249
Sex (male), n(%)	97,532 (57.3)	88,171 (57.2)	9,361 (57.6)	0.397
JCS score at admission, n (%)				<0.001
0	82,353 (48.4)	74,857 (48.6)	7,496 (46.1)	
1~3	67,436 (39.6)	60,732 (39.4)	6,704 (41.2)	
10~300	20,505 (12.0)	18,450 (12.0)	2,055 (12.6)	
Severity at admission, n(%)				0.022
independent	22,577 (13.3)	20,517 (13.3)	2,060 (12.7)	
dependent	147,717(86.7)	133,522(86.7)	14,195(87.3)	
Treatment approach				
Intravenous thrombolysis, n(%)	10,919 (6.4)	9,836 (6.4)	1083 (6.7)	0.175
Endovascular intervention, n(%)	7,393 (4.3)	6641 (4.3)	752 (4.6)	0.064
Transfer to other facilities, n(%)	54,744 (32.1)	49,392 (32.1)	5,352 (32.9)	0.026
Length of hospital stay, median days [IQR]	19 [11–35]	19 [11–36]	18 [11–32]	<0.001

JCS: Japan Coma Scale, ADL: Activities of Daily Living, IQR: Interquartile Range.

### Interrupted time series analysis for changes in ischemic stroke hospitalized cases

**Fig 1:**
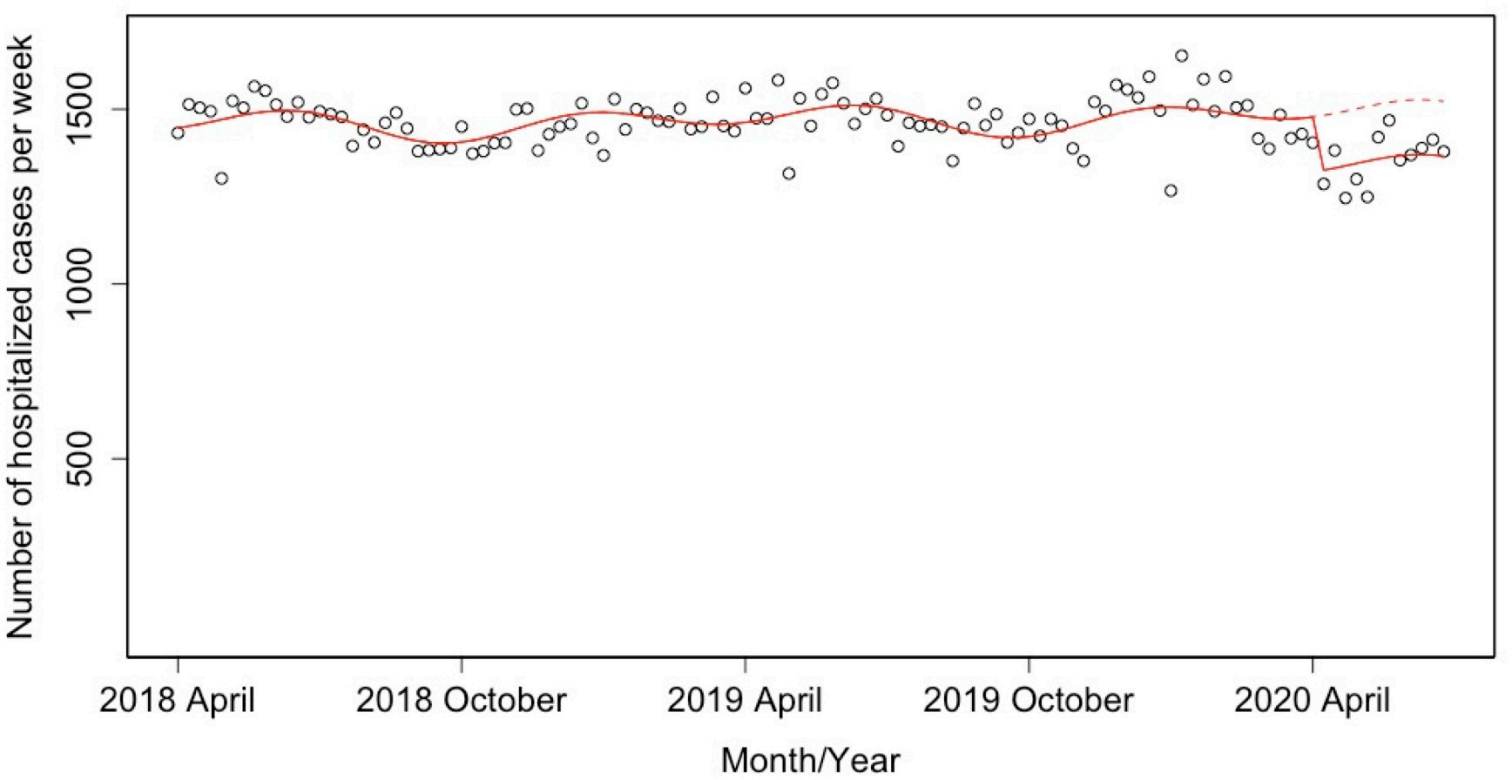
Result of the interrupted time series analysis for the weekly number of inpatients with ischemic stroke between April 1 2018 and June 27 2020. Solid lines indicate the predicted trend based on a model, and dashed lines indicate the predicted trend based on a model in the scenario without the state of emergency.

**Fig 2:**
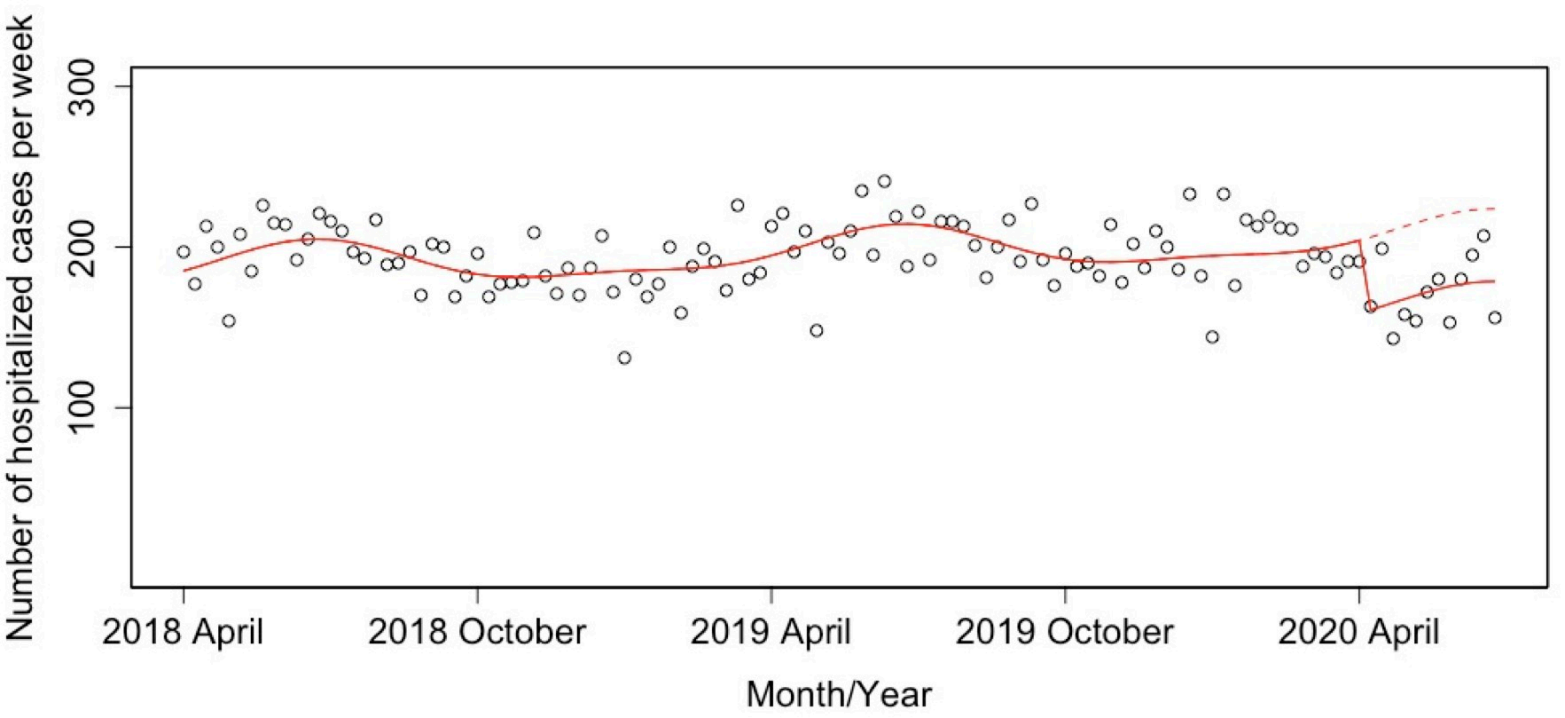
Result of the interrupted time series analysis for the weekly number of independent group inpatients with ischemic stroke between April 1 2018 and June 27 2020. Solid lines indicate the predicted trend based on a model, and dashed lines indicate the predicted trend based on a model in the scenario without the state of emergency.

**Fig 3:**
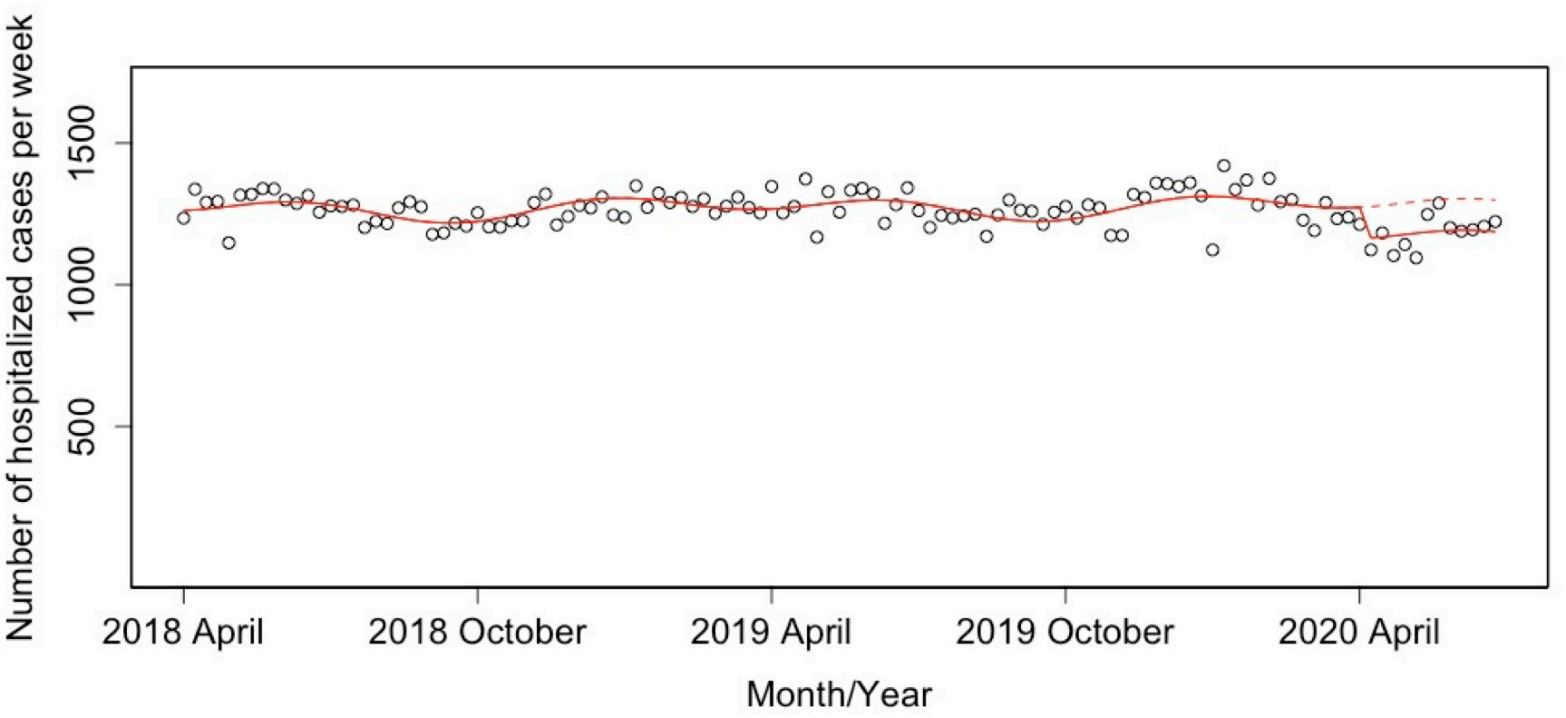
Interrupted time series analysis for the weekly number of dependent group inpatients with ischemic stroke between April 1 2018 and June 27 2020. Solid lines indicate the predicted trend based on a model, and dashed lines indicate the predicted trend based on a model in the scenario without the state of emergency.

The ITS analysis showed a significant decrease in the weekly number of ischemic stroke hospitalized cases (estimated decrease: −157 cases; 95% confidence interval (CI): −209 to −104) (Fig 1), which corresponds to −10.4% (95% CI: −13.6 to −7.1) after the declaration of state of emergency for the COVID-19 pandemic. Figs 2 and 3 show the weekly numbers of hospitalized cases for independent and dependent groups with the result of the ITS analysis. The ITS analysis revealed a significant decrease of 45 independent cases (95% CI: −61 to −30) and 111 dependent cases (95% CI: −155 to −68). The proportion of decline in the independent group (−21.3%; 95% CI: −26.0 to −16.2) was larger than that in the dependent group (−8.6%; 95% CI: −11.7 to −5.4).

### Impact on treatment administration during the COVID-19 pandemic

The ITS analysis showed that there was no significant change in the proportions of inpatient cases receiving intravenous thrombolysis (level change −5.0%; 95% CI: −13.0 to 3.8) and endovascular intervention (level change −6.3%; 95% CI: −15.8 to 4.3) (S2 and S3 Figs).

## Discussion

In this study, we used a large-scale administrative database to evaluate the impact of the COVID-19 pandemic on the weekly number of inpatients due to ischemic stroke in Japan. The main finding of our study showed that: 1) hospitalized cases due to ischemic stroke significantly decreased after the declaration of state of emergency in Japan; 2) the proportion of decline in the independent group was larger than that in the dependent group; and 3) no significant decrease was observed in weekly proportions of cases receiving intravenous thrombolysis or endovascular intervention.

The decrease in the number of inpatient cases due to ischemic stroke, especially in non-severe cases, is consistent with a previous report [12]. There are two possible mechanisms for the decline of inpatient cases. First, patients with ischemic stroke, especially those without severe symptoms such as paralysis and disturbance of consciousness, may be hesitant to seek medical services for fear of exposure to COVID-19. In other fields, including paediatrics and cardiology, patients were found to be reluctant to seek medical evaluation to avoid contact with COVID-19 in hospital [9, 17]. Another possible explanation for the decrease in the number of inpatients is the lack of contact with others resulting from stay-at-home and social distancing practices. Physical distancing measures could deprive patients of witnesses to the onset of ischemic stroke [10]. In cases of acute stroke, it is not uncommon for friends and neighbours to request emergency help [18].

Even in emergency conditions such as the COVID-19 pandemic, appropriate evaluation and management of stroke is necessary. Any delay in the medical evaluation of ischemic stroke cases, even mild cases, may result in severe consequences, including long-term disability, pneumonia due to dysphagia, and the early recurrence of stroke. For example, dual antiplatelet therapy within 24 hours of the onset of symptoms decreases the risk of recurrent stroke in patients with high-risk transient ischemic attack (TIA) or minor ischemic stroke [19]. Governments need to recognize that stroke patients with mild symptoms may be reluctant to seek medical evaluation during a pandemic, particularly where social distancing and stay-at-home practices are being recommended, and should provide appropriate information about the symptoms of ischemic stroke. Those with symptoms suggesting stroke should be encouraged to seek appropriate medical evaluation immediately, even if the symptoms are mild.

The length of hospital stay decreased after the declaration of state of emergency, especially among the inpatient cases who transferred to other facilities. The reason for the decrease in the length of hospital stay was unclear. The decrease in inpatient cases after the declaration of state of emergency might enable early transfer to other facilities for rehabilitation.

Our study revealed that no significant decline was observed in the weekly proportions of cases receiving intravenous thrombolysis or endovascular intervention. Previous studies showed the proportion of intravenous thrombolysis and thrombectomy decreased in China and Spain where their medical services were inundated [20, 21]. Our result may reflect the relative extent of damage to the health care system by the COVID-19 pandemic. We assume the resources of the health care system were properly provided in stroke care in Japan, where the number of COVID-19 cases was relatively small in this period, and the extent of damage was less than in other countries [20, 21].

Our study has several limitations. First, our DPC data did not include ischemic stroke onset time. Consequently, the delay from symptom onset to hospital arrival was not analysed in detail. In addition, our research included only hospitalized cases. The change from hospitalization to outpatient management during the COVID-19 pandemic might lead to an overestimate of the impact. However, patients with acute ischemic stroke should be admitted to a stroke unit and most patients were hospitalized [22, 23]. Despite these limitations, our research provides important information on the impact of the COVID-19 pandemic on stroke patients.

## Conclusion

Our results show a marked reduction in weekly hospital admissions due to ischemic stroke after the state of emergency for the COVID-19 pandemic was declared in Japan, using large-scale administrative data. Our ITS analyses identified that the proportion of decline in non-severe cases was larger than that in severe cases.

## Supporting information

S1 Fig Flow of case selection. (TIF)

S2 Fig The result of interrupted time series analysis for weekly proportions of inpatient cases receiving intravenous thrombolysis between April 1 2018 and June 27 2020. Solid lines indicate the predicted trend based on a model, and dashed lines indicate the predicted trend based on a model in the scenario without the state of emergency.
(TIF)

S3 Fig The result of the interrupted time series analysis for weekly proportions of inpatient cases receiving endovascular intervention between April 1 2018 and June 27 2020. Solid lines indicate the predicted trend based on a model, and dashed lines indicate the predicted trend based on a model in the scenario without the state of emergency.
(TIF)

S1 Table The total number, characteristics, and time-sensitive treatment approaches of inpatient cases with ischemic stroke who did not transfer to other facilities before and after the declaration of state of emergency. (DOCX)

S2 Table The total number, characteristics, and time-sensitive treatment approaches of inpatient cases with ischemic stroke who transferred to other facilities before and after the declaration of state of emergency. (DOCX)
