# Supplementary figures and images for: Hospitalization for ischemic stroke was affected more in independent cases than in dependent cases during the COVID-19 pandemic: An interrupted time series analysis
Source: PLoS One. 2021 Dec 17;16(12):e0261587. doi: 10.1371/journal.pone.0261587 (PMC8682905; doi:10.1371/journal.pone.0261587)

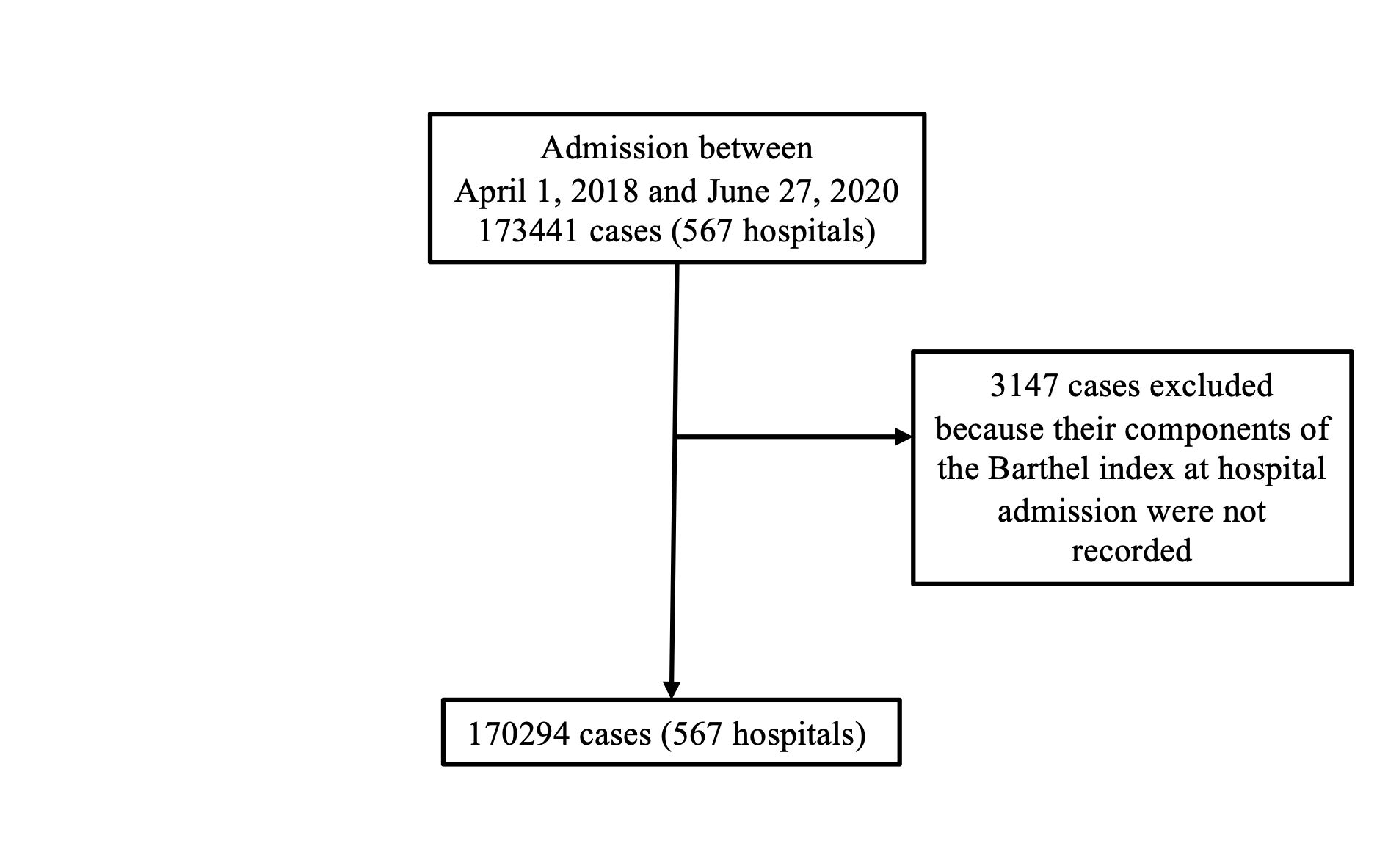

Supplement: S1 Fig — (TIF) [file pone.0261587.s001.tif]

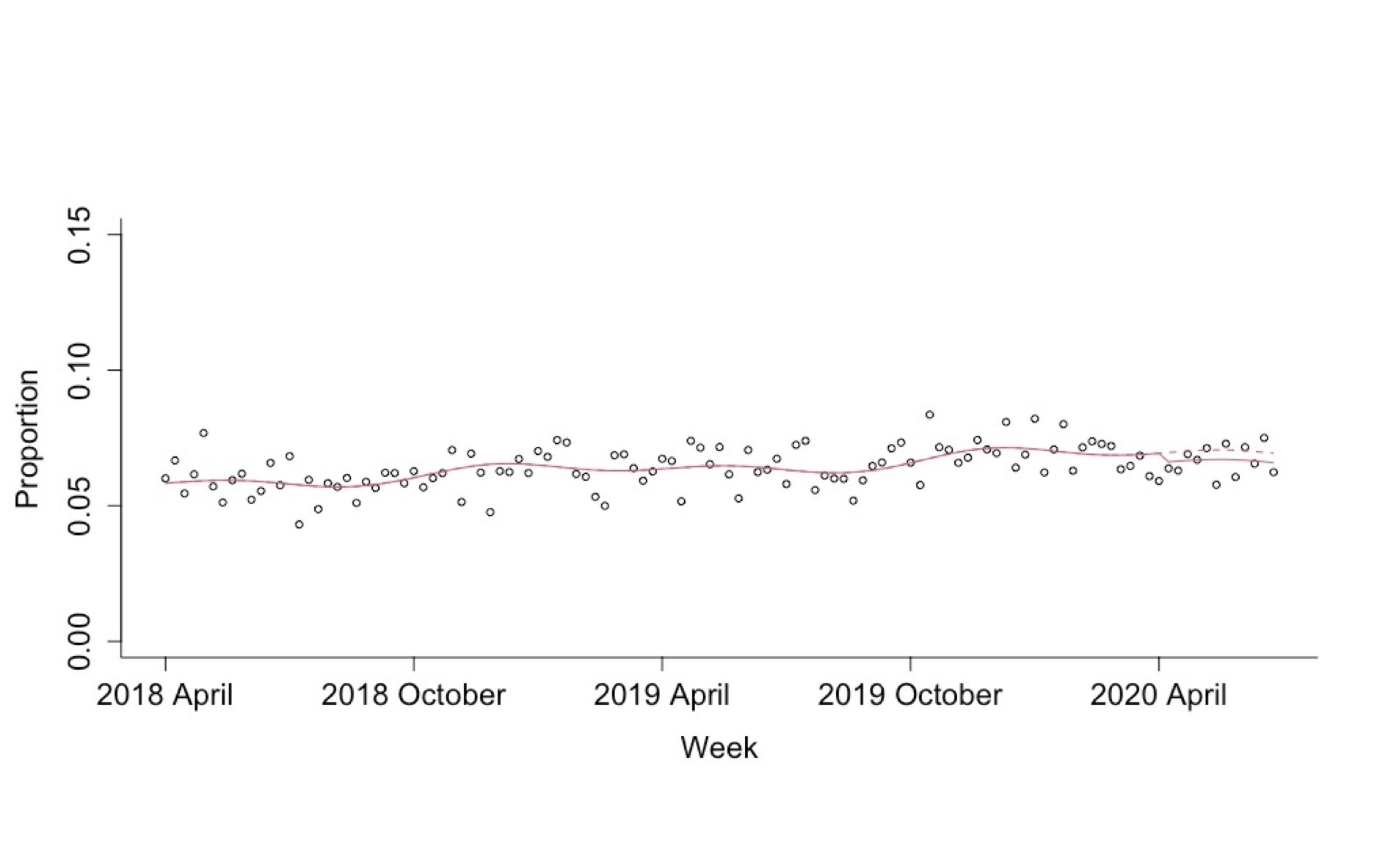

Supplement: S2 Fig — Solid lines indicate the predicted trend based on a model, and dashed lines indicate the predicted trend based on a model in the scenario without the state of emergency. (TIF) [file pone.0261587.s002.tif]

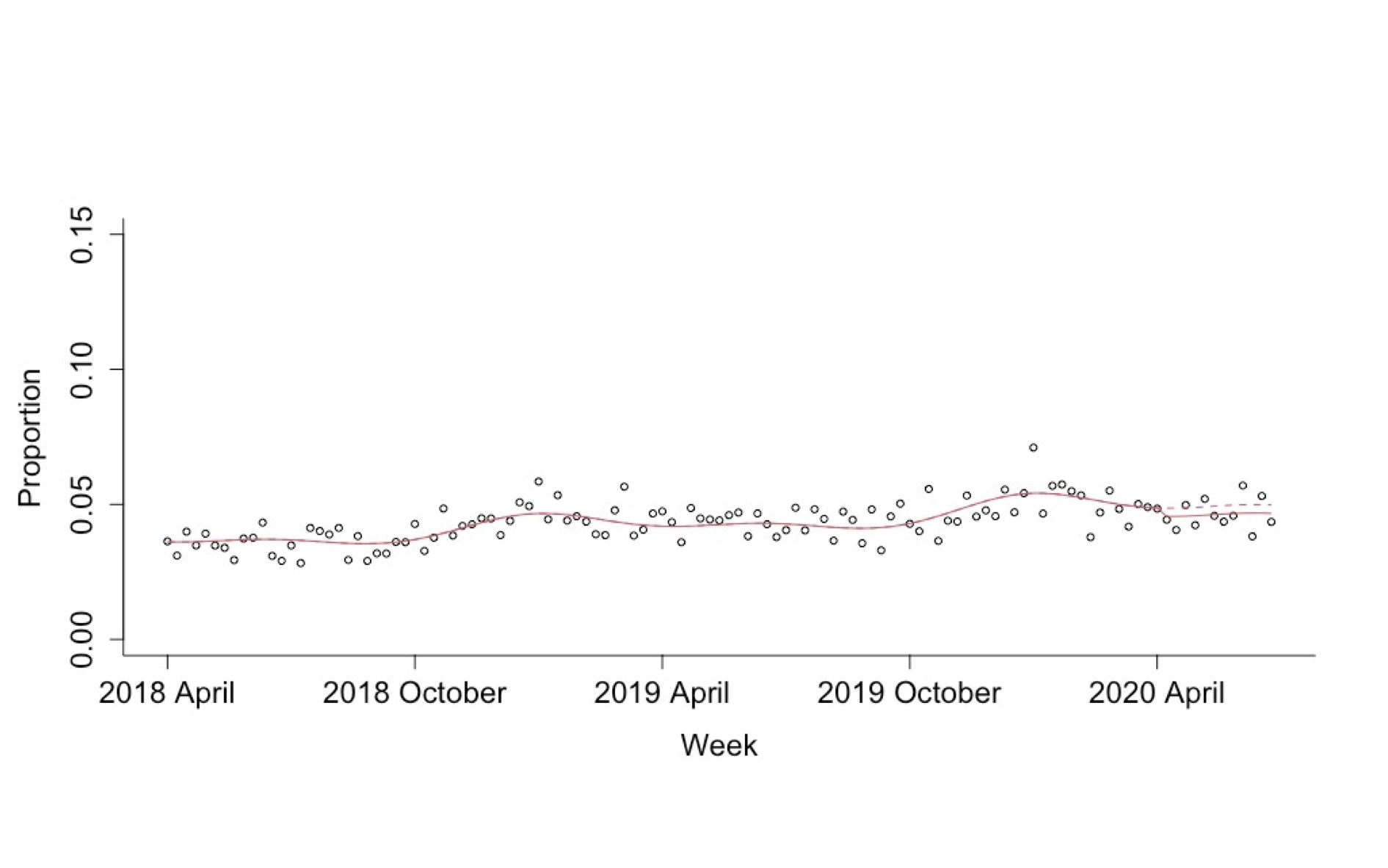

Supplement: S3 Fig — Solid lines indicate the predicted trend based on a model, and dashed lines indicate the predicted trend based on a model in the scenario without the state of emergency. (TIF) [file pone.0261587.s003.tif]
