# Supplementary material for: Hospitalization for ischemic stroke was affected more in independent cases than in dependent cases during the COVID-19 pandemic: An interrupted time series analysis
Source: PLoS One. 2021 Dec 17;16(12):e0261587. doi: 10.1371/journal.pone.0261587 (PMC8682905; doi:10.1371/journal.pone.0261587)
Supplement: S1 Table — (DOCX) [file pone.0261587.s004.docx]

|  | Before the  declaration of state of emergency | After the  declaration of state of emergency | p |
| --- | --- | --- | --- |
| Number of cases | 104,647 | 10,903 |  |
| Age, y, median [IQR] | 77 [68-84] | 77 [69-84] | 0.328 |
| Sex (male), n(%) | 61,123 (58.4) | 6,444 (59.1) | 0.165 |
| JCS score at admission, n (%) |  |  | <0.001 |
| 0 | 59,288 (56.7) | 5,864 (53.8) |  |
| 1~3 | 36,407 (34.8) | 4,052 (37.2) |  |
| 10~300 | 8952 (8.6) | 987 (9.1) |  |
| Severity at admission, n(%) |  |  | 0.075 |
| independent | 18,231 (17.4) | 1,825 (16.7) |  |
| dependent | 86,416 (82.6) | 9,078 (83.3) |  |
| Treatment approach |  |  |  |
| Intravenous thrombolysis, n(%) | 5,111 (4.9) | 607 (5.6) | 0.002 |
| Endovascular intervention, n(%) | 2,751 (2.6) | 311 (2.9) | 0.176 |
| Length of hospital stay, median days [IQR] | 15 [10-31] | 15 [10-28] | <0.001 |

JCS: Japan Coma Scale, ADL: Activities of Daily Living, IQR: Interquartile Range
