# Supplementary material for: Hospitalization for ischemic stroke was affected more in independent cases than in dependent cases during the COVID-19 pandemic: An interrupted time series analysis
Source: PLoS One. 2021 Dec 17;16(12):e0261587. doi: 10.1371/journal.pone.0261587 (PMC8682905; doi:10.1371/journal.pone.0261587)
Supplement: S2 Table — (DOCX) [file pone.0261587.s005.docx]

|  | Before the  declaration of state of emergency | After the  declaration of state of emergency | p |
| --- | --- | --- | --- |
| Number of cases | 49,392 | 5,352 |  |
| Age, y, median [IQR] | 80 [72-86] | 80 [72-86] | 0.749 |
| Sex (male), n(%) | 27,048 (54.8) | 2,917 (54.5) | 0.729 |
| JCS score at admission, n (%) |  |  | 0.22 |
| 0 | 15,569 (31.5) | 1,632 (30.5) |  |
| 1~3 | 24,325 (49.2) | 2,652 (49.6) |  |
| 10~300 | 9,498 (19.2) | 1,068 (20.0) |  |
| Severity at admission, n(%) |  |  |  |
| independent | 2,286 (4.6) | 235 (4.4) | 0.452 |
| dependent | 47,106 (95.4) | 5,117 (95.6) |  |
| Treatment approach |  |  |  |
| Intravenous thrombolysis, n(%) | 4,725 (9.6) | 476 (8.9) | 0.117 |
| Endovascular intervention, n(%) | 3,890 (7.9) | 441 (8.2) | 0.362 |
| Length of hospital stay, median days [IQR] | 27 [19-40] | 24 [17-36] | <0.001 |

JCS: Japan Coma Scale, ADL: Activities of Daily Living, IQR: Interquartile Range
